# Electrolytes Play a Role in Detecting Cisplatin-Induced Kidney Complications and May Even Prevent Them—Retrospective Analysis

**DOI:** 10.3390/medicina59050890

**Published:** 2023-05-06

**Authors:** Bahauddeen M. Alrfaei, Abdulaziz O. Almutairi, Alaa A. Aljohani, Hajar Alammar, Abdulaziz Asiri, Yahya Bokhari, Feda S. Aljaser, Manal Abudawood, Majed Halwani

**Affiliations:** 1 Cellular Therapy and Cancer Research Department, King Abdullah International Medical Research Center (KAIMRC), King Saud bin Abdulaziz University for Health Sciences, Riyadh 14611, Saudi Arabia; 2 College of Medicine, King Saud bin Abdulaziz University for Health Sciences, Ministry of National Guard-Health Affairs, Riyadh 14611, Saudi Arabia; 3 College of Pharmacy, King Saud bin Abdulaziz University for Health Sciences, Ministry of National Guard-Health Affairs, Riyadh 14611, Saudi Arabia; 4 Faculty of Applied Medical Sciences, University of Bisha, 255, Al Nakhil, Bisha 67714, Saudi Arabia; 5 Department of AI and Bioinformatics, King Abdullah International Medical Research Center (KAIMRC), King Saud bin Abdulaziz University for Health Sciences, Riyadh 11481, Saudi Arabia; 6 Department of Health Informatics, College of Public Health and Health Informatics, King Saud bin Abdulaziz University for Health Sciences, Riyadh 11426, Saudi Arabia; 7 Department of Clinical Laboratory Sciences, College of Applied Medical Sciences, King Saud University, Riyadh 12371, Saudi Arabia; 8 Nanomedicine Department, King Abdullah International Medical Research Center (KAIMRC), King Saud bin Abdulaziz University for Health Sciences, Riyadh 11481, Saudi Arabia

**Keywords:** cisplatin, electrolyte, cancer, hypomagnesemia, hypokalemia, kidney failure

## Abstract

*Background and Objective*: Cisplatin is a chemotherapy drug used to treat several types of malignancies. It is a platinum-based compound that interferes with cell division and DNA replication. Cisplatin has been associated with renal damage. This study evaluates the early detection of nephrotoxicity through routine laboratory tests. *Materials and Methods:* This is a retrospective chart review based on the Saudi Ministry of National Guard Hospital (MNGHA). We evaluated deferential laboratory tests for cancer patients treated with cisplatin between April 2015 and July 2019. The evaluation included age, sex, WBC, platelets, electrolytes, co-morbidities and interaction with radiology. *Results*: The review qualified 254 patients for evaluation. Around 29 patients (11.5%) had developed kidney function abnormality. These patients presented with abnormally low magnesium 9 (31%), potassium 6 (20.7%), sodium 19 (65.5%) and calcium 20 (69%). Interestingly, the whole sample size had abnormal electrolytes presenting magnesium 78 (30.8%), potassium 30 (11.9%), sodium 147 (58.1%) and calcium 106 (41.9%). Some pathological features were detected, such as hypomagnesemia, hypocalcemia and hypokalemia. In addition, infections that needed antibiotics were dominant in patients treated with cisplatin alone, representing 50% of this group. *Conclusions*: We report that an average of 15% of patients with electrolyte abnormalities develop renal toxicity and reduced function. Moreover, electrolytes may serve as an early indicator for renal damage as part of chemotherapy complication. This indication represents 15% of renal toxicity cases. Changes in electrolyte levels have been reported with cisplatin. Specifically, it has been linked to hypomagnesemia, hypocalcemia and hypokalemia. This study will help reduce the risk of dialysis or the need for kidney transplant. It is also important to manage any underlying conditions and control patients’ intake of electrolytes.

## 1. Introduction

Cisplatin, which also called cis-diamminedichloridoplatinum(II), is a chemotherapy agent based on platinum [1]. It is used to suppress cancer cell division and expansion by inhibiting DNA synthesis through the formation of DNA cross-links. Cisplatin can also bind to two adjacent guanines on the same strand of DNA, producing an intra-strand cross-linking and breakage. Cisplatin is usually given intravenously (into a vein) as part of a combination of drugs. It is used to treat as many 20% of all malignancies, such as ovarian, testicular, cervical, lung, bladder, head and neck [2,3]. 

Cisplatin is associated with dose-dependent complications, including nausea, vomiting, hypomagnesemia, peripheral neuropathy, bone marrow suppression and hearing loss. Two of the most common cisplatin side effects are ototoxicity and nephrotoxicity. The ototoxicity (toxicity or damage to the ear) shows bilateral and irreversible symptoms of tinnitus and hearing loss [4]. The nephrotoxicity (toxicity or damage to the kidneys) shows renal injury or loss of kidney function [3]. The mechanism of cisplatin action on the kidneys is poorly understood. Several routes are suggested to cause nephrotoxicity and kidney failure: tubular epithelial cell toxicity, vasoconstriction in the renal microvasculature and pro-inflammatory response [5]. A study led by Olsen documented renal disorder in 14% of testicular cancer patients who received cisplatin compared to 8% of patients who received radiotherapy [5]. Ethnicity seems to play a role when cisplatin is used, since a report shows that African American patients have a higher risk than white patients of developing nephrotoxicity [6]. In addition, a high dose of cisplatin (more than 50 mg m^−2^ body surface area) was also reported to increase side effects, especially nephrotoxicity [7]. Additionally, a published meta-analysis of 13 randomized clinical trials (RCTs) that included a total of 2829 patients showed that a 1.7% increase in treatment-related mortality was detected for cisplatin-based therapy compared to 0.8% for radiotherapy alone. Therefore, the study concluded that cisplatin treatment in cancer patients has an increased risk of mortality over radiotherapy alone [8]. Cisplatin is known to lead to symptoms such as infection, anemia, feeling sick, loss of appetite, changes in taste, kidney problems, hearing disorders, bruising and bleeding [9,10]. Cisplatin symptoms and toxicity have been reviewed in Saudi cancer patients. We report the incidence and patterns among Saudi patients, which may reflect the situation in the Gulf area and Middle East in general. Our aim in this study is to investigate the prevalence and significance of the side effects associated with cisplatin therapy in cancer patients in Saudi Arabia, which represents the Gulf region.

## 2. Statistical Analysis

Analysis included descriptive statistical frequency, data presentation, mean and standard deviation. Significance was assessed using the chi-square test and parametric or non-parametric binomial tests where appropriate. The level of significance was set at 0.05 for all tests (*p* < 0.05) and this was the criterion to consider the results statistically significant.

## 3. Method

This is a retrospective chart review based on the Saudi Ministry of National Guards Hospital and Health Affairs (MNGHA), a tertiary hospital in Riyadh. All medical records of patients diagnosed with cancer who received cisplatin treatment between April 2015 and July 2019 were included. Inclusion criteria were patients receiving cisplatin alone or in combination with chemotherapy or radiation. We used a non-probability consecutive sampling technique. The patients were followed up for at least two months based on physicians’ recommendation. The evaluation included age, sex, WBC, platelets, electrolytes, co-morbidities, tumor size and interaction with radiology. Exclusion criteria were patients with no clinical treatment information or patients who had undergone cancer treatment prior to admission to MNGHA. 

## 4. Results

Overall, 260 Saudi patients found for the period between 2015 and 2019. The number of qualifying patients based on the criteria was 254. Gender was found to be an insignificant controller for the cisplatin data. In this study, the number of males was 124, while there were 130 females. The BMIs were calculated to represent normal (56.7%) and overweight (43.3%) scores, which were insignificant for developing nephrotoxicity (*p* = 0.631). The median score for BMI was 23. In addition, the median age for the study was 46 years old. The patients’ marital status was recorded, with 150 (59.3%) married, 75 (29.6%) single and 28 (11.1%) other patients. The patient cohort included 19.7% treated with cisplatin alone while 80.3% were treated with cisplatin in combination with other chemotherapies. The cohort included pediatric patients, who represented 13.4% of the total. The patients’ demographic characteristics are detailed in Table 1.

The data present 27 different cancers that have been treated with cisplatin. The most frequent ones are lymphoma general (20%), breast cancer (9.8%), nasopharyngeal cancer (7.5%), stomach cancer (6.3%), neuroblastoma (5.8%), medulloblastoma (5%), testicular cancer (5%), lung cancer (4.7%), Hodgkin lymphoma (4.3%), and others (<4%); see Table 2. The most common cancer treated with cisplatin in our institution (MNGHA center) is lymphoma, which represent 20% of all treated cases (Table 2). In addition, Hodgkin lymphoma represents 4.3% of all treated cases (Table 2). It is worth noting that immunotherapy such as CAR T-cell therapy for lymphoma has been introduced to MNGHA, but the outcome is still under assessment. Therefore, CAR *t*-cell immunotherapy is not included in this study. The second most common cancer treated with cisplatin in our study is breast cancer, which represents around 10% of all cases (Table 2). The rest of the cases in the cisplatin cohort at a level above 4% are nasopharyngeal cancer (7.5%), stomach cancer (6.3%), neuroblastoma (5.8%), medulloblastoma (5%) and lung cancer (4.7%). Interestingly, metastasis represents 46.9% of all cases treated with cisplatin, Table 2.

There are 24 different chemotherapies used in the MNGHA center in combination with cisplatin. Any medication used in more than 10% of cases was listed as frequent therapy, such as gemcitabine, etoposide and cytarabine; see Table 3. In addition, four medications were used in between 5 and 9% of cases, which are vincristine, rituximab, docetaxel and capecitabine. Although limited significance was expected, medications used in less than 5% of cases in our cohort are reported in Table 3. Some patients suffered from infections and needed treatment with antibiotics, representing 35.2% of all cases. Moreover, patients treated with cisplatin alone suffered from infections at a ratio of around 10% of all cases but 50% of cisplatin-alone cases.

Kidney toxicity was reported in 11.5% of all cases. Among the affected patients, 69% had low calcium abnormality, while only 20% had low potassium abnormality, representing the upper and lower limit, respectively. Total abnormalities which are above or below the normal range were documented as follows: calcium 41.9%, sodium 58.1%, magnesium 30.8% and potassium 11.9% (Table 4).

Co-morbidities (Table 5) considered for this study represent 27.7% of cases, which include hypertension (high blood pressure), diabetes mellitus (inadequate control of blood sugar), deep vein thrombosis (blood clot formation in a vein, mostly in lower limbs), pulmonary embolism (a blood clot that blocks and stops blood flow to an artery in the lungs), dyslipidemia (imbalance or high levels of lipids such as cholesterol, low-density lipoprotein cholesterol and triglycerides), end-stage renal disease (kidneys stop functioning on a permanent basis), chronic kidney disease (damaged kidney that cannot filter blood properly), atrial fibrillation (abnormal electrical impulses start firing in the atria), systemic lupus erythematosus (autoimmune disease in which the immune system attacks its own tissues), acute kidney injury (kidneys suddenly stop working properly), multiple sclerosis (immune-mediated inflammatory disease that attacks myelinated axons, causing disruption to central nervous system work flow) and liver cirrhosis (liver function prevented by scarring (fibrosis) of the liver). Most of the patients, 72%, had no co-morbidities documented (Table 5). In addition, radiation therapy was not applied in 79.8% of patients. Even though 20.2% of cisplatin patients in our study were exposed to radiation, more than 41% had abnormal platelets. The contribution of co-morbidities, which represent 27.7% of cases, to platelet abnormality (41.9%) was not significant (*p* = 0.5), (Table 5).

In total, 36.6% of patients in the cohort suffered from large tumors >5 cm, while 64.8% had tumors smaller than 5 cm. Abnormal ranges of platelets represented 15.6% (excess) and 26% (deficiency) of the whole cohort. White blood cell (WBC) distribution was measured as 17% above normal and 44% below normal range (Table 5).

## 5. Discussion

We evaluated renal damage in 254 patients who had received cisplatin as part of their cancer therapy between 2015 and 2019. We observed an overall incidence of kidney toxicity of 11.5%. 

Looking at the study demographic characteristics, we found no significant predictor relevant to gender or nationality. Moreover, kidney toxicity was not detected in any of the pediatric cases, even though they represent 13.4% of the cohort treated with cisplatin. This suggests that pediatric patients have more tolerance to the chemotherapy treatments than adults. Nonetheless, it is important to consider that this is a relatively small sample size to draw solid conclusions for pediatric patients. Other studies have observed ototoxicity (severe hearing loss), which was not investigated in this study [11,12]. Furthermore, BMI’s contribution to cisplatin response severity was not confirmed statistically (*p* = 0.631).

This study extracted cancer cases that were associated with cisplatin treatment. These cases represented 27 different cancers, of which lymphoma represented 20% of the cohort. The other cancers were present in the cohort at levels from 10 to 4%. This study did not intend to study a particular cancer, but rather trace the complications arising from cisplatin therapy. Furthermore, as part of the treatment protocols, we documented 24 different chemotherapies used in combination with cisplatin. These treatments were used by physicians relevant to particular malignancies and standards. It is worth noting that some of these drugs, such as etoposide, rituximab and capecitabine, were reported to contribute to kidney toxicity [13,14,15]. However, cisplatin has a higher frequency based on the literature [16,17]. Cisplatin has been associated with changes in electrolyte levels. Specifically, it has been linked to hypomagnesemia (a condition caused by a low level of magnesium in the body), hypocalcemia (a condition in which the level of calcium in the blood is lower than normal) and hypokalemia (a condition in which the level of potassium in the blood is lower than normal) [18]. 

Cisplatin affects the tubulointerstitial components of the kidney with relative sparing of glomeruli. Therefore, glomerular filtration rate and creatinine level may not be the best indicators of nephrotoxicity. As electrolytes are mostly reabsorbed in the tubular system, cisplatin-induced tubular atrophy would result in electrolyte disturbance [19,20]. Magnesium wasting is a common electrolyte abnormality in cisplatin-treated individuals, which could be associated with preserved glomerular filtration rate (GFR) readings and could persist for months [21,22]. An earlier study demonstrated that the levels of hypomagnesemia were positively correlated with cumulative doses of cisplatin [21]. In addition, hyponatremia and renal salt wasting are due to proximal tubule dysfunction. Increased sodium levels in the distal tubules trigger a decrease in renal blood flow and GFR through the macula densa-induced tubular–glomerular feedback loop. Dysfunction in the distal tubule and loops of Henle fails to compensate for the reabsorption and contributes to the increased excretion [23]. Other electrolyte abnormalities are hypokalemia and hypocalcemia, which in addition to tubular injury, also commonly result from magnesium deficiency, which plays an essential role in their regulation [22].

The mechanisms of the underlying renal injury are mainly secondary to cisplatin accumulation in the kidney [24]. The renal system clears cisplatin via glomerular filtration and secretion through the tubular transporters Ctr1 and organic cation transporter 2 (OCT2) [25,26]. Genetic variations in the transporters are correlated with the degree of nephrotoxicity [27,28]. Additionally, cisplatin is conjugated to glutathione by glutathione transferase and then converted by gamma-glutamyl transpeptidase (GGT- mediated biotransformation into a more potent component [29,30]. Other mechanisms are related to cisplatin cytotoxicity as a chemotherapeutical agent. Cisplatin molecules bind directly to nuclear and mitochondrial DNA, leading to DNA synthesis arrest or the induction of apoptotic pathways, most prominently the caspase-9–dependent pathway [26]. In addition to mitochondrial injury, oxidative stress from the increased production of reactive oxygen species in the treated kidneys is a significant component of apoptosis cascade activation [31]. Moreover, cisplatin induces p300, which binds to NF-κB and regulates the signaling pathway through acetylation. These activities stimulate functional and histological injury, increasing oxidative stress, inflammation and immunological processes [26].

Cancer stem cells (CSCs) were reported to be resistant to conventional chemotherapy and radiation [32]. Cisplatin was found to increase enrichment for CSCs instead of reducing it. In addition, cisplatin stimulates CSCs through a transcriptional increase in PIK3CA, which makes multiple subunits, one of which contributes to PI3K/AKT production [33]. Cisplatin induces the expression of the pluripotency genes OCT4, SOX2 and NANOG. It was also documented that a lack of NF-κB, TNFα and PIK3CA make some stem cells susceptible to cisplatin [33]. Thus, anti-NF-κB and PIK3CA are probably needed in combination with cisplatin therapy.

In this study, we reviewed electrolytes such as sodium, calcium, magnesium and potassium as early indicators for kidney dysfunction. We found that in the kidney failure group, abnormally low levels of calcium 69%, sodium 65.5%, magnesium 31% and potassium 20% were present before severe kidney dysfunction. The data suggest that calcium and sodium level abnormalities may work as the best indicators in this context. Deficiency in both the electrolytes shown predicted 65% of the nephrotoxicity cases. However, conventional diagnostic and clinical methods will still be in place for confirmation and proper medical response. Looking at the whole data, we see an exaggeration of calcium and sodium abnormality, representing 41.9% and 58.1% of cases, respectively. While kidney toxicity cases represent only 11.5% of all cases, better indicators are needed. Therefore, we suggest using all electrolytes, calcium 69%, sodium 65.5%, magnesium 31% and potassium 20%, as indicators that give a narrow window for kidney dysfunction prediction. 

Even though cisplatin treatment alone represents 20% of all cases, infection in patients treated with cisplatin alone was documented in around 10% of all cases. This means that 50% of patients treated with cisplatin alone developed infection. Similarly, cisplatin is reported in the literature to lead to infection, anemia, feeling sick, loss of appetite, changes in tongue, kidney problems, hearing disorders, bruising and bleeding [9,10].

Hydration was suggested to postpone or decrease the nephrotoxic effect of cisplatin. However, its efficacy is still limited [2]. Hydration decreases cisplatin’s half-life and reduces renal transit time, thereby limiting drug accumulation in the kidneys [34]. Recent evidence recommends low-volume hydration along with magnesium and potassium supplementation for patients taking low and intermediate cisplatin doses, and adding mannitol to hydration, a hypertonic diuretic, for high cisplatin doses [34,35]. Another study found that electrolyte replacement therapy with magnesium, potassium and calcium during cisplatin treatment, besides hydration with normal saline, had a protective effect against nephrotoxicity and increased survival, according to Kaplan–Meier analysis [36]. There are multiple less-established preventive strategies [37]. For example, OCT2 inhibitors such as Cimetidine could be used to decrease tubular entry and renal accumulation [38]. Additionally, since oxidative stress is an established mechanism of cisplatin toxicity, the administration of antioxidants, such as Dimethylthiourea (DMTU), has shown some positive progress. However, this could interfere with the antitumor effects of the medication [39]. On the other hand, biotransformation inhibitors mostly spare tumor cells and reduce renal toxicity [37]. For instance, thiol group providers such as Amifostine inhibit the formation of the cisplatin–glutathione conjugate, thereby preventing GGT metabolism into potent forms [20].

Amifostine and sodium thiosulfate also protect against platinum-induced hearing loss [40,41]. Amifostine was approved for cisplatin renal protection in ovarian cancer [42]. Another example is glutathione, which competitively inhibits GGT [34]. Anti-inflammatory medications are another class of drugs studied to address cisplatin’s toxic side effects without reducing the anti-neoplastic aspects by targeting the cytokines involved in the pathogenesis, such as TNFα and TLR4 [20,25].

## 6. Limitations

Even though this study reports an association between electrolyte abnormality and renal failure, other chemotherapies used in this study need to be considered. This study reports that only 20% of patients were exposed to cisplatin alone, while 80% of patients had combination therapy including cisplatin. This affects the reliability of the data as some of the nephrotoxicities may be caused by combination therapy rather than cisplatin alone. The cause-and-effect relationship cannot be considered in this case. However, 17% of the kidney failure group were from the cisplatin-alone cohort, which is a significant number. Additionally, this study is a retrospective review, which adds another layer to the limitation.

## 7. Conclusions

We reported herein a possibility of using electrolyte deficiency as an early indicator for renal failure. We encountered limited early and late nephrotoxicity. Importantly, nephrotoxicity was not the main dose-limiting toxicity. This study will help reduce the risk of dialysis or the need for kidney transplant when better patient management and patient’ awareness are practiced. Our results emphasize the importance of close monitoring and additional replacement of water and electrolytes as needed. Patients should be advised to increase electrolyte intake or prescribed electrolytes as a precaution. On the other hand, patients who suffer from abnormally high electrolytes should take a prescribed drug to reduce electrolyte imbalance. More effort needs to be made to generate a consistent method of measuring and reporting chemotherapy-induced nephrotoxicity, which would be a valuable contribution to the literature.

## Figures and Tables

**Table 1:** Demographic characteristics of cisplatin patients treated between 19 April 2015 and 17 July 2019. (N.A.: not applicable).

No.	Characteristics		Patients No. (%)
1	Gender	Male	124 (48.81%)
		Female	130 (51.4%)
2	Nationality and Marital status	Saudi	253 (100%)
		Single	75 (29.6%)
		Married	150 (59.3%)
		Divorced	2 (0.8%)
		N.A.	26 (10.3%)
3	Age (2–84 years) Median 46 years	Pediatric patients	34 (13.4%)
		Adults	220 (86.6%)
4	BMI	Overweight	110 (43.3%)
		Normal weight	144 (56.7%)
5	Cisplatin	Alone	50 (19.7%)
		In combination	204 (80.3%)

**Table 2:** Different cancers treated with cisplatin in MNGHA. (*)Cases not included in the total due to duplication.

No.	Cisplatin-Treated Tumors	Number of Cases	Total Percentage
1	Testicular cancer	13	5%
2	Lymphoma Hodgkin	53	20%
		11 *	4.3%
3	Breast cancer	25	9.8%
4	Neuroblastoma	15	5.8%
5	Medulloblastoma	13	5%
6	Stomach cancer	16	6.3%
7	Nasopharyngeal	19	7.5%
8	Lung cancer	12	4.7%
9	Others (<10 cases per cancer) seminoma, osteosarcoma, buccal esophageal cancer, bladder cancer, tongue cancer, renal cancer, lung cancer, external ear cancer, pancreatic cancer, gallbladder cancer, cervical cancers, neuroendocrine carcinoma, cholangiocarcinoma, hepatoblastoma, multiple myeloma, immature teratoma, choriocarcinoma, adenocarcinoma	87	34.4%
	Metastasis	119 *	46.9%
	Total	253	100%

**Table 3:** Chemotherapy drugs used along with cisplatin.

No.	Medication Used with Cisplatin	Number of Cases	Total Percentage
1	Gemcitabine	45	17.8%
2	Etoposide	85	33.6%
3	Cytarabine	29	11.5%
4	Bleomycin	9	3.6%
5	Capecitabine	15	5.9%
6	Paclitaxel	7	2.8%
7	Ifosfamide	4	1.6%
8	Docetaxel	14	5.5%
9	Cetuximab	3	1.2%
10	Pemetrexed	5	2%
11	Doxorubicin	9	3.6%
13	Pemetrexed	6	2.4%
14	Cyclophosphamide	6	2.4%
15	Vincristine	14	5.5%
16	Methylprednisolone	2	0.8%
17	Lomustine	4	1.6%
18	Rituximab	14	5.5%
19	Epirubicin	9	3.6%
20	Fluorouracil	10	4%
21	Bortezomib	3	1.2%
22	Crizotinib	1	0.4%
23	Irinotecam	2	0.8%
24	Trastuzumab	1	0.4%
25	Antibiotics Cisplatin	89 25	35.2% 9.8%

**Table 4:** Laboratory tests predict kidney complications. (N.A.: not applicable).

No.	Parameters	Normal	Abnormal
1	Sodium (Na)	106 (41.9%)	147 (58.1%)
2	Potassium (K)	223 (88.1%)	30 (11.9%)
3	Magnesium (Mg)	175 (69.6%)	78 (30.8%)
4	Calcium (Ca)	147 (58.1%)	106 (41.9%)
5	Kidney function Abnormally low Mg Abnormally low K Abnormally low Na Abnormally low Ca	224 (88.5%)	29 (11.5%) Toxicity
		N.A.	9 (31%) 6 (20.7%) 19 (65.5%) 20 (69%)

**Table 5:** Risk factors might contribute to disease complications. cm: centimeter; µL: microliter.

No.	Parameter	No	Yes
1	Co-morbidity	183 (72.3%)	70 (27.7%)
2	Interaction with radiology	202 (79.8%)	51 (20.2%)
3	Tumor size > 5 cm	163 (64.8)	90 (35.6%)
4	Abnormal platelets >400 per µL <150 per µL	147 (58.1%) 213 (84.2%) 187 (73.9%)	106 (41.9%) 40 (15.7%) 66 (26%)
5	Abnormal WBC >11,000 per µL <4000 per µL	98 (38.7%) 210 (83%) 141 (55.7%)	155 (61.3%) 43 (17%) 112 (44.3%)

## Data Availability

The dataset collected and analyzed in the present study is obtainable from the corresponding author on reasonable request.

## Abbreviations

Ministry of National Guards Hospital and Health Affairs (MNGHA), white blood cells (WBCs), randomized clinical trials (RCTs), body mass index (BMI), not applicable (N.A.), sodium (Na), potassium (K), magnesium (Mg), calcium (Ca), centimeter (cm); microliter (µL), cancer stem cells (CSCs), gamma-glutamyl transpeptidase (GGT), glomerular filtration rate (GFR), organic cation transporter 2 (OCT2), chimeric antigen receptor (CAT T-cell). NF-κB: nuclear factor kappa light chain-enhancer of activated B cells. PIK3CA: phosphatidylinositol-4,5-bisphosphate 3-kinase catalytic subunit alpha. PI3K/AKT: phosphoinositide 3-kinases/protein kinase B. OCT-4: octamer-binding transcription factor 4. SOX2: sex-determining region Y-box 2. NANOG: homeobox protein NANOG. TNFα: tumor necrosis factor alpha.
